# Case investigation and reactive case detection for malaria elimination in northern Senegal

**DOI:** 10.1186/1475-2875-12-331

**Published:** 2013-09-17

**Authors:** Megan Littrell, Gnagna Dieng Sow, Algaye Ngom, Mady Ba, Balla Mbacke Mboup, Yakou Dieye, Boniface Mutombo, Duncan Earle, Richard W Steketee

**Affiliations:** 1 PATH Malaria Control and Evaluation Partnership (MACEPA), Seattle, WA, USA; 2 PATH Malaria Control and Evaluation Partnership (MACEPA), Dakar, Senegal; 3 Richard Toll District Medical Office, Richard Toll, Senegal; 4 Senegal National Malaria Control Programme, Dakar, Senegal; 5 Saint-Louis Regional Medical Office, Saint-Louis, Senegal; 6 PATH Malaria Control and Evaluation Partnership (MACEPA), Lusaka, Zambia

**Keywords:** Malaria, Surveillance, Case investigation, Reactive case detection, Elimination, Senegal

## Abstract

**Background:**

Given progress in malaria control in recent years, many control programmes in sub-Saharan Africa will soon be required to strengthen systems for surveillance in order to further drive transmission to zero. Yet few practical experiences are available to guide control programmes in designing surveillance system components in low transmission, pre-elimination, and elimination phases.

**Methods:**

A malaria case investigation programme was piloted for 12 weeks in 2012 in Richard Toll district of northern Senegal. Malaria infections (N = 110) were identified through facility-based passive case detection and investigated within three days. Rapid diagnostic tests (RDT) and a brief questionnaire were administered to 5,520 individuals living within the index case compound or within five neighbouring compounds.

**Results:**

In comparison with family and neighbours, index cases were more likely to be male, age 15–49, and to report travel within the past 15 days that entailed an overnight stay. Twenty-three (0.4%) of family/neighbours were RDT-positive. Potential risk factors for infection among family and neighbours were examined, including: sex, age, occupation, travel history, bed net usage, and residence (index *vs* neighbouring compound). Adjusting for all factors, relative risk (RR) of infection was associated with residence in the index case household (RR = 3.18, p < 0.05) and recent travel, including travel to Dakar (RR = 19.93, p < 0.001), travel within the region (RR = 9.57, p < 0.01), and to other regions in Senegal (RR = 94.30, p < 0.001). Recent fever among RDT-positive family/neighbours was uncommon (30%). Modifications to testing criteria were examined to optimize the efficiency of secondary case investigations in this population. Limiting blood testing to residents of the index case compound and neighbours with recent travel or fever would have identified 20/23 (87%) of the infections through testing 1,173 individuals. Information on the remaining three infections suggests that additional screening for boarding school attendees may facilitate identification of all cases.

**Conclusions:**

The primary risk factor for malaria infection in the low transmission district of Richard Toll is travel. Additional intervention and monitoring strategies to target travellers at risk of malaria infection are needed in this region. Optimizing case investigation with specific targeted testing and treatment of at-risk family and neighbours strengthens the systems needed for continued progress towards malaria elimination in northern Senegal.

## Background

Malaria case investigation and reactive case detection are critical components in malaria programme pre-elimination and elimination phases. Given progress in malaria control in recent years, many control programmes in sub-Saharan Africa will soon be required to strengthen systems for surveillance and response in order to further drive transmission to zero. Yet few practical experiences are available to guide control programmes in designing surveillance system components in low transmission, pre-elimination, and elimination phases. This paper presents results from a 2012 pilot case investigation and reactive case detection activity undertaken in a low-transmission district of northern Senegal. Findings from this pilot project can guide control programme measures to modify and strengthen surveillance for elimination in similar contexts.

### Malaria control progress in Senegal

Senegal achieved rapid and sustained progress in controlling malaria in recent years. Parasitaemia measured among children under five in the first national Malaria Indicator Survey with malaria blood testing in 2008 was 5.7% [1] and fell to 2.9% in 2010–11 [2]. Between 2005 and 2008–09, all-cause under-five mortality dropped from 121 to 72 deaths per 1,000 live births [1,2]. Reduction in malaria morbidity and mortality was achieved through policy change and rapid scale-up of interventions facilitated by strategic planning and organization, and increased funding [3].

National figures on malaria burden in Senegal mask substantial sub-national variation in transmission. Parasitaemia in children under five ranges from 0% in the northern Saint-Louis region to 13.5% in the south-western region of Kedougou [2]. To facilitate targeting of interventions, the National Malaria Control Programme (NMCP) stratified the country according to disease burden, defined by the 2009 annual incidence rate. Updates to stratification have not been possible since 2009 due to a national data retention strike in which public health workers withhold routine data from national health information systems. Districts are classified using routine data on passively-detected malaria cases as either: 1) low, less than five cases per 1,000 population; 2) moderate, between five and 15 cases per 1,000 population; and 3) high, more than 15 cases per 1,000 population. Fifty-three districts in central and southern Senegal are classified as moderate or high transmission areas. Districts in the Dakar region are classified as moderate or high transmission and are at particular risk due to flood patterns. The 11 districts in the north and two additional districts in western and southern Senegal are classified as low transmission. Moderate and high transmission districts require further intensification of prevention and control measures. Northern districts classified as ‘low’ are prioritized in the NMCP’s 2011–2015 National Strategic Plan for pre-elimination activities to identify, investigate, and treat all cases [4].

### Surveillance for malaria elimination: case investigation and reactive case detection

Identifying, investigating and treating all malaria infections is achieved through a strong system of surveillance and appropriate response. During the control phase, surveillance data on spatial and temporal trends in cases are used to target resources and track progress over time. In the presence of relatively high incidence, it is neither feasible nor efficient to closely examine and react to each individual malaria case [5].

As malaria incidence declines, it is increasingly feasible and important to collect case data that can inform appropriate action. Moving towards elimination, case data collection is expanded to include more detailed demographic information and travel history via a standard questionnaire. This information is used to classify a malaria infection as locally acquired (autochthonous) or imported. Officials and health workers are subsequently notified at various levels to trigger further action. In a low transmission setting, passively detected cases can be used to identify population groups that are sources of infection [6]. An infection identified through passive case detection – or an index case – can be used to trigger additional case detection activities targeting the population group associated with the passively identified case. Through this reactive case detection, additional secondary infections may be identified among the people living in close proximity to the index case and/or people who share a workplace or occupational risk factor [7].

### Operationalizing reactive case detection

Protocols and terminology used to describe the work of finding and treating secondary infections vary widely [8]. A key variation pertains to inclusion criteria for malaria blood testing. Reactive case detection can refer to initial screening for fever/history of recent fever and limiting blood testing to febrile/recently febrile individuals, or testing all individuals regardless of recent febrile illness [6]. Reactive case detection here refers to the later.

The scale of the investigation – in terms of definition of the population at risk for secondary infection – is a key consideration with implications for human and financial resources required to be successful. Reactive investigations typically consider all people living within the index case household. The extent to which other neighbours are considered varies. Strategies to define households for investigation have included targeting people living within a specific radius of the index case, or targeting a specific number of proximate people or households for follow-up [9]. WHO guidance suggests covering a large population, given that the flight range of the *Anopheles* mosquito is typically 1–2 km [6]. In practice, decisions regarding an appropriate catchment have been arbitrary due to the lack of evidence around what is most effective [10]. Standard operating procedures recently implemented by countries aiming for elimination include Sri Lanka in 2009 and Swaziland in 2010, both identifying a 1-km radius for reactive case investigation [11,12]. However, a 1-km radius has proven logistically challenging and appears unsustainable in Swaziland [13]. A malaria elimination feasibility assessment undertaken in Zanzibar, focused on modeling second-generation cases per 1,000 person years, identified need for active case detection among approximately 100 neighbouring households around each identified case to prevent re-emergence of malaria [14].

Although countries are beginning to gain operational experience, and the WHO released surveillance guidelines for elimination settings in 2012, to date there is insufficient evidence around the most effective and efficient ways to operationalize reactive case detection across various epidemiological settings [10,15,16]. The pilot activity reported here was undertaken to inform scale-up of such activities in low-transmission settings of Senegal and similar contexts in sub-Saharan Africa.

## Methods

### Surveillance site – Richard Toll District, northern Senegal

**Figure 1:**
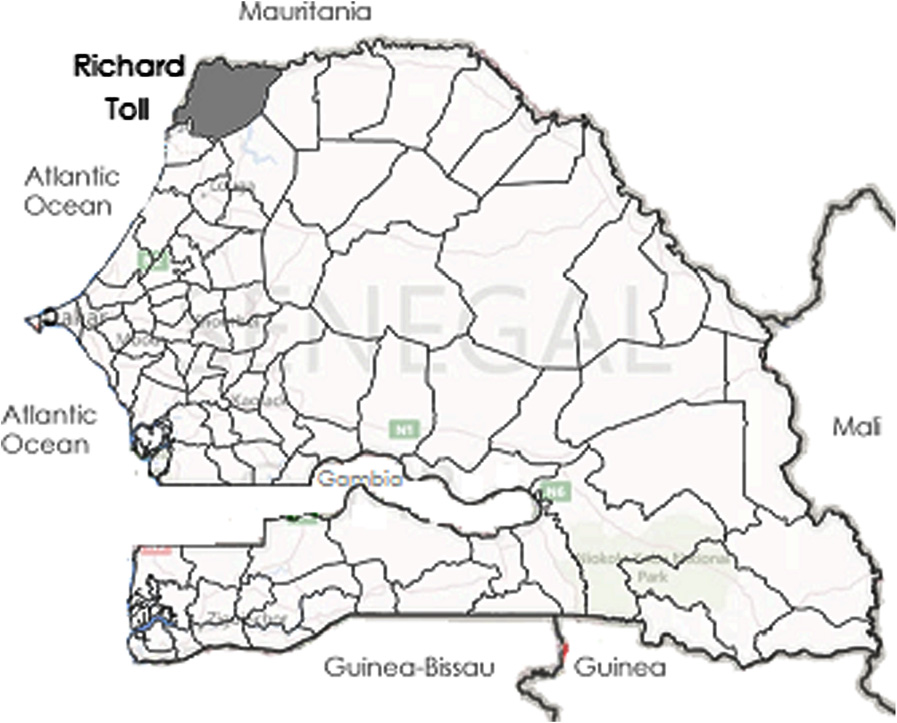
Richard Toll district in Northern Senegal.

Richard Toll is a low-transmission district located in the northern Saint-Louis region of Senegal (Figure 1), where parasite prevalence among children under five in 2008 and 2010–2011 population-based surveys was 0% [1,2]. The Senegal River runs through the Saint-Louis region, along the northern border with Mauritania. Richard Toll is the site of major irrigation schemes tied to the Senegal River as well as Senegal’s largest lake, Lake Guiers. A dam was built in the 1980s across the Taouvey tributary stream through which Senegal River floods flow to Lake Guiers. Irrigation networks facilitate agriculture in Richard Toll, including large-scale sugar cane production by the Senegalese Sugar Company (SSC). The SSC was established in 1972 and employs approximately 6,000 permanent staff and 3,000 seasonal workers between November to June.

The 2012 population of Richard Toll District was approximately 153,000 residing within an area of 2,912 sq km. About half the population lives in urban areas in major settlements including Richard Toll town. The people of Richard Toll district are predominately Muslim, and major ethnic groups include Wolof (60%) and Pulaar (35%).

Malaria transmission in Richard Toll occurs towards the end of and immediately following the July to October rainy season. Due to its vast irrigation surfaces, the district has a secondary seasonal transmission peak in April. Vector control coverage is high in Richard Toll. The district was targeted with support from the US President’s Malaria Initiative for indoor residual spraying (IRS) from 2007 to 2012. The 2010–2011 Demographic and Health Survey (DHS) (with oversampling of Richard Toll District) found high coverage of IRS (76%), and nearly all households (97%) either owned at least 1 insecticide-treated bed net (ITN) or had been sprayed in the previous 12 months [2].

### Case investigation and reactive case detection

**Figure 2:**
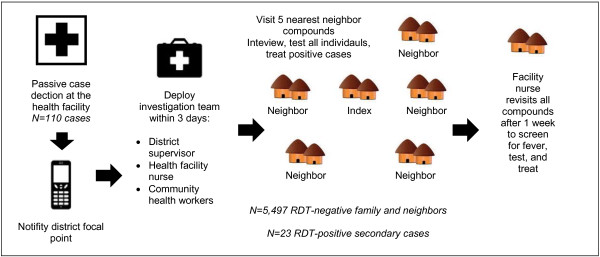
Case investigation and reactive case detection procedures.

The case investigation programme piloted in northern Senegal was a partnership between the NMCP, the Malaria Control and Evaluation Partnership (MACEPA) at PATH, and district officials, public health facilities, and community health workers in Richard Toll District. Infections identified through facility-based passive case detection were documented by a health worker and information was sent to the district via a telephone call or mobile phone text message. With very rare exceptions due to network problems, the district was notified of a malaria case the same day that the case was identified at the health facility. Within three days of notification, a team was deployed to community level to conduct the detailed case investigation and reactive case detection. Broad stakeholder participation, district ownership, and dedicated and reliable resources for field investigations ensured that all cases reported to the district were investigated within three days. The core investigation team was comprised of a nurse from the reporting health facility, one of four district health supervisors, community health worker(s) from the index case community, and a MACEPA field coordinator. NMCP focal points and/or the MACEPA monitoring and evaluation coordinator accompanied the team on a routine basis to provide supportive supervision. The team divided the work of visiting the index case compound and the five neighbouring compounds. Given settlement patterns in Richard Toll district, this typically entailed identifying individuals living within a 100- to 150-m radius of the index case compound in urban areas (e.g., Richard Toll town) and a 300- to 500-m radius of the index case compound in other areas (Figure 2).

The geographic scale of reactive investigation activities can be driven by a number of considerations. WHO notes that factors such as recent local transmission and vector, environment and climate factors that favour transmission (receptivity) are among those to be considered when determining the scale of investigation activities [6]. The Richard Toll district pilot was conducted during a known peak transmission season, in an environment with large irrigation surfaces and known presence of *Anopheles* mosquitoes. The investigation catchment area of five neighbouring compounds was selected given that local transmission was entirely possible, but balanced by practical considerations of what was operationally feasible to achieve with available resources and within the desired time frame (i.e., investigation initiated within three days of notification). Operationally, this defined catchment area meant that approximately 48 to 54 people would be interviewed and tested during each investigation.

Within each compound (comprised of all nuclear households affiliated with a compound head), basic demographic information, recent febrile illness history, and recent travel history (travel that entailed sleeping at least one night away from home within the previous 15 days) were collected for each individual. Rapid diagnostic tests (RDT, SD Bioline Malaria Ag Pf) were administered to all people, regardless of fever history. RDTs may miss infections detectable by more sensitive methods [17], and initial evidence suggests that submicroscopic infections may contribute to transmission in such settings [18]. While additional information is needed to establish the extent to which highly sensitive assays are needed for this type of work, this programme pilot in northern Senegal employed RDTs because they are available and feasible for field use and programme action. All infections identified by rapid diagnostic testing were treated according to national policy with artemether-lumefantrine. The participation rate for blood testing and data collection among family and neighbors was 98%. High participation was facilitated by: 1) advance cascade sensitization whereby the health facility contacted the village health committee and requested that a committee member notify affected compounds on the evening prior to the investigation team visit; 2) initial compound visits and booking appointments for follow-up with absent members; and 3) return visits to the compound the same or next day. Upon completion of initial case investigation activities, follow-up visits were made to all investigated compounds after one week to identify any new fever cases for blood testing. These follow-up visits are an important layer to case investigation, providing an extension to the window for identifying secondary infections associated with an index case. In this pilot activity, no additional infections were identified through these follow-up visits.

**Figure 3:**
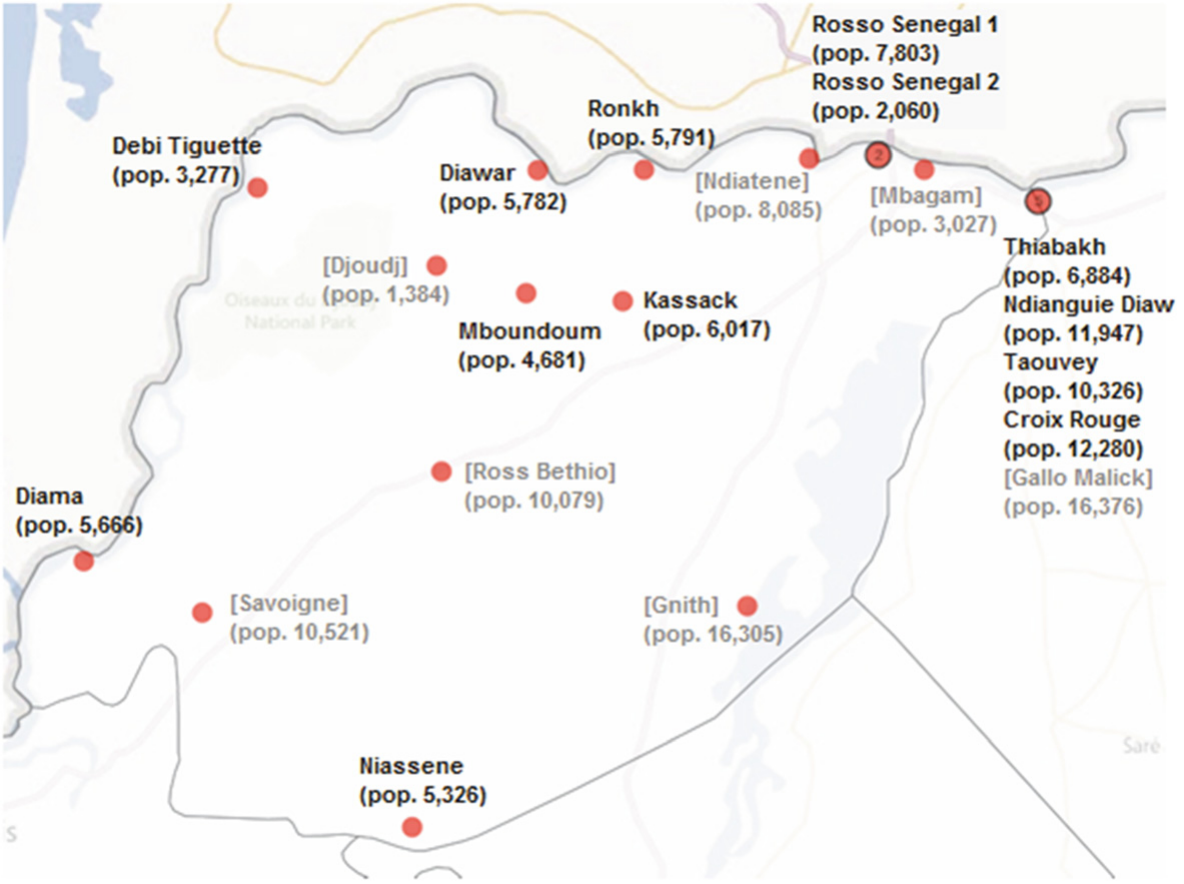
**Location and catchment population sizes of health facilities in Richard Toll district.** Health facilities in brackets were not reporting during the pilot period due to a national data retention strike.

Twelve health posts and one health centre participated in case investigation and reported to district level, despite a data retention strike in which health workers at public facilities were withholding routine data from national health information systems. Seven additional public health facilities were trained in case investigation, however they did not participate in pilot activities due to the data retention strike. Health facility participation in pilot activities therefore effectively covered 57% of the district population (Figure 3).

Training for the Richard Toll district health officer, public health nurses, and 322 community health workers was conducted in July 2012. The pilot operation was conducted for 12 weeks beginning in September 2012. The case investigation protocol was reviewed by the National Malaria Control Programme and its Steering Committee and deemed to be programme work and evaluation and therefore exempt from full research review by the Institutional Review Board. Formal individual consent was not required given that data were generated through standard public health surveillance activities and were de-identified before analysis.

### Data collection and analysis

A total of 5,630 investigation forms were completed and entered in Epi Info™ (Centers for Disease Control and Prevention, Atlanta, GA, USA). Data were analysed in Stata 12.1 (© StataCorp, College Station, TX, USA). Descriptive statistics were used to create a profile of basic demographic and travel history information for index cases, RDT-positive family and neighbors (secondary cases), and RDT-negative family and neighbours. Unadjusted risk ratios were estimated for potential factors associated with a positive RDT result among family and neighbours. Factors with significant unadjusted risk ratios were included in a single model to produce adjusted estimates. Risk ratios were estimated using Poisson working models with robust sandwich variance estimator.

## Results

Within the catchment area of 13 reporting health facilities with an approximate population of 87,840 people, 110 cases were identified through passive case detection, resulting in a period (12-week high transmission season) incidence of 1.25 cases per 1,000 population. A total of 5,520 individuals living within index and neighbouring compounds were evaluated and tested, of which 23 (0.4%) were RDT-positive. Facilities located within Richard Toll town cover 47% of the total population located within facility catchment areas. Sixty-six percent of index cases were identified in health facilities in Richard Toll town and investigation of these cases identified 87% of all RDT-positive household members or neighbours (Table 1).

**Table 1:** Passive malaria case detection index cases and RDT results among family and neighbours investigated around each case, within each health facility catchment area

	**Total population**		**Index cases**		**Family and neighbours of the index case**			
	**n**	**%**	**n**	**(%)**	**n**	**(%)**	**n**	**(%)**
**Within Richard Toll town:**								
Thiabakh health centre	6,884	(7.8)	21	(19.1)	3	(13.0)	820	(14.9)
Ndiangue Diaw	11,947	(13.6)	22	(20.0)	3	(13.0)	1,253	(22.8)
Taouvey	10,326	(11.8)	16	(14.6)	10	(43.5)	604	(11.0)
Croix Rouge	12,280	(14.0)	14	(12.7)	4	(17.4)	929	(16.9)
Total town	41,437	(47.2)	73	(66.4)	20	(87.0)	3,606	(65.6)
**Outside of Richard Toll town:**								
Debit Tiguette	3,277	(3.7)	1	(0.9)	0	(0.0)	0	(0.0)
Diama	5,666	(6.5)	9	(8.2)	1	(4.4)	402	(7.3)
Diawar	5,782	(6.6)	2	(1.8)	0	(0.0)	227	(4.1)
Kassack-Sud	6,017	(6.8)	1	(0.9)	0	(0.0)	53	(1.0)
Mboundoum	4,681	(5.3)	1	(0.9)	0	(0.0)	92	(1.7)
Niasséne	5,326	(6.1)	14	(12.7)	2	(8.7)	485	(8.8)
Ronkh	5,791	(6.6)	1	(0.9)	0	(0.0)	115	(2.1)
Rosso Senegal 1	7,803	(8.9)	7	(6.4)	0	(0.0)	404	(7.4)
Rosso Senegal 2	2,060	(2.3)	1	(0.9)	0	(0.0)	40	(0.7)
Total outside of town	46,403	(52.8)	37	(33.6)	3	(13.0)	1,891	(34.4)
**Total**	87,840	(100.0)	110	(100.0)	23	(100.0)	5,497	100.0

**Figure 4:**
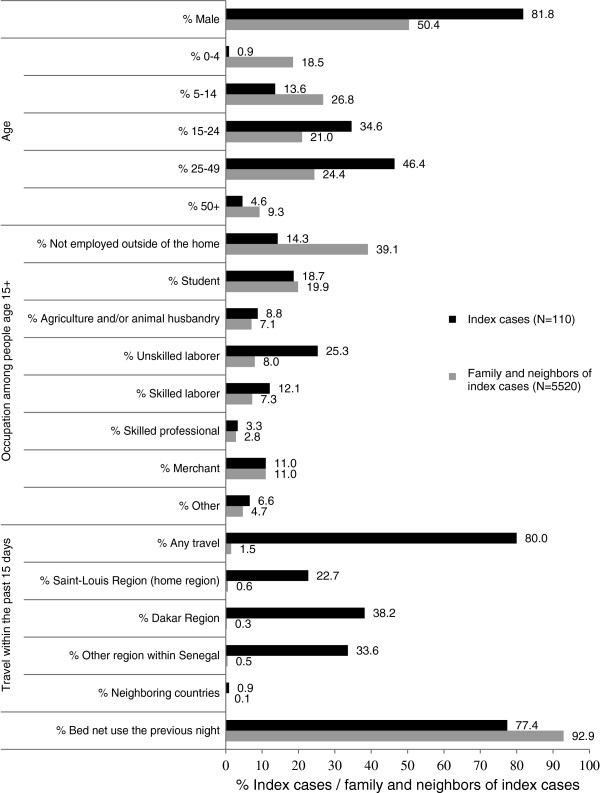
**Characteristics of index cases and neighbours of the index case.** Characteristics of index cases identified through facility-based case detection in comparison with family and neighbours of the index case – people living within index or neighbouring compounds.

Figure 4 presents a profile of index cases identified through facility-based passive case detection in comparison with family and neighbours of the index case. In comparison with family and neighbours, index cases were disproportionately male (82% *vs* 50% of family/neighbours), and between the ages of 15 and 24 (35% *vs* 21%) and 25–49 (46% *vs* 25%). Within adult age groups, index cases were more likely to be employed outside of the home (86% *vs* 61%), and more likely to work in unskilled labour (25% *vs* 8%) or skilled labour (12% vs 7%). Most family/neighbours reported using a bed net the previous night (93%) compared with 77% of index cases. Index cases were far more likely than members of the household or neighbours to report travel that entailed sleeping at least one night away from home in the previous 15 days (80% *vs* 2%), including travel within Saint-Louis (home) region (23%), Dakar region (38%), and other regions in Senegal (34%) (Figure 4).

The prevalence of malaria infection (RDT-positive) among family and neighbours of index cases was 0.4%. Prevalence was significantly higher among people living in the index case household (1.3%) compared to people living in neighbouring households (0.2%), and higher among men (0.6%) compared with women (0.2%). Prevalence was significantly higher among people with recent travel history (15.7%) compared with people without travel history (0.2%). Across recent travel destinations, the percentage of people testing RDT-positive varied by destination: Saint-Louis region, 3.1%; Dakar region, 10.5%; and other regions in Senegal, 33.3%. Prevalence was also higher among people who reportedly did not use a bed net the previous night (1.3%) compared with those who did report net use (0.4%). Adjusting for all other factors, relative risk (RR) of infection was significantly higher among family and neighbours with recent travel history, including travel within the Saint-Louis region (RR = 9.57, 95% CI = 2.32-39.45), travel within the Dakar region (RR = 19.93, 95% CI = 4.02-98.83), and travel to other regions in Senegal (RR = 94.30, 95% CI = 34.24-259.69). Residence within the index case compound *versus* neighbouring compound also had a significant adjusted association with RDT-positivity (RR = 3.18, 95% CI = 1.21-8.39) (Table 2). Among family and neighbours who had a positive RDT, only 30% reported recent fever. This compares with a 1% fever history among people that tested negative for malaria.

**Table 2:** **Unadjusted and adjusted**^**1 **^**relative risk of positive RDT result among family and neighbours of index cases across residence, sex, age, occupation, recent travel history, and bed net use**

	**N**	**RDT-positive**		**Unadjusted RR**	**Adjusted RR**^**1**^
		**n (%)**		**(95% CI)**	**(95% CI)**
**Residence – compound**					
Index case	1,076	14	(1.3)	6.42 (2.79-14.80)^***^	3.18 (1.21-8.39)^*^
Neighbouring	4,444	9	(0.2)	ref	ref
**Sex**					
Male	2,735	17	(0.6)	2.88 (1.14-7.29)^*^	1.58 (0.55-4.52)
Female	2,779	6	(0.2)	ref	ref
**Age**					
0-4	1,011	1	(0.1)	ref	--
5-14	1,468	6	(0.4)	4.13 (0.50-34.28)	--
15-24	1,151	6	(0.5)	5.27 (0.64-43.71)	--
25-49	1,336	8	(0.6)	6.05 (0.76-48.33)	--
50+	511	1	(0.2)	1.98 (0.12-31.57)	--
**Occupation among people age 15+**					
Not employed outside of the home	1,019	2	(0.2)	ref	--
Student	518	4	(0.8)	3.93 (0.72-21.40)	--
Agriculture and/or animal husbandry	186	0	(0.0)	0.0	--
Unskilled labourer	209	2	(1.0)	4.87 (0.69-34.41)	--
Skilled labourer	191	1	(0.5)	2.67 (0.24-29.27)	--
Skilled professional	74	0	(0.0)	0.0	--
Merchant	286	3	(1.1)	5.34 (0.90-31.82)	--
Other	123	1	(0.8)	4.14 (0.38-45.35)	--
**Travel within the previous 15 days**					
Any travel	83	13	(15.7)	85.16 (38.44-188.67)^***^	---
No travel	5,437	10	(0.2)	ref	---
Travel within Saint-Louis region (home region)	32	1	(3.1)	7.80 (1.08-56.10)^*^	9.57 (2.32-39.45)^**^
None within the region	5,488	22	(0.4)	ref	ref
Travel within Dakar region	19	2	(10.5)	27.57 (6.95-109.47)^***^	19.93 (4.02-98.83)^***^
None within the region	5,501	21	(0.4)	ref	ref
Travel within other region in Senegal	30	10	(33.3)	140.77 (67.01-295.72)^***^	94.30 (34.24-259.69)^***^
None within other regions	5,490	13	(0.2)	ref	ref
Travel to neighbouring countries	3	1	(33.3)	83.59 (15.99-436.96)^***^	omitted
None to neighbouring countries	5,517	22	(0.4)	ref	---
**Bed net use the previous night**					
No	387	5	(1.3)	3.64 (1.36-9.76)^*^	1.90 (0.58-6.18)
Yes	5,077	18	(0.4)	ref	ref
**Total**	5,520^2^	23	(0.4)	--	--

*p < 0.05, **p < 0.01, ***p < 0.001.

1 Full model (observations = 5,458) containing residence, sex, recent travel, and bed net use. Travel to neighbouring countries was omitted due to instability with small n (n = 3 with travel history to neighbouring countries).

2 Missing data: Sex, n = 6; Age, n = 43; Occupation among people age 15+, n = 392; Bed net use, n = 60.

Table 3 summarizes scenarios for applying more restrictive criteria to the scope of reactive case detection. Limiting blood testing to residents of the index compound would have entailed testing 1,076 individuals and would have yielded 14/23 (61%) of the infections. Testing all index compound members plus screening neighbours and testing only those with recent travel or recent fever would have entailed testing 1,173 individuals and would have yielded 20/23 (87%) of RDT positives (Table 3). The remaining three infections that would not have been identified using this screening strategy were male students, including one university student and two secondary school students. While it cannot be determined from the data collected, it is possible that these students did not have recent travel history, but were boarding at school in another region.

**Table 3:** Number of people tested with an RDT, and number of RDT-positive infections and the fraction of all infections that would be identified through variations on the scope of reactive case detection

	**Number tested with an RDT**	**Number RDT-positive**	**Fraction of all RDT-positive infections**
Index compound only	1,076	14	14/23 (60.9)
**Index compound plus people living in 5 neighbouring compounds with:**			
Any recent travel	1,120	20	20/23 (87.0)
Any recent travel, or residing in a compound with someone that recently travelled	1,382	20	20/23 (87.0)
Recent fever	1,133	18	18/23 (78.3)
Recent fever, or residing in a compound with someone that recently had fever	1,337	18	18/23 (78.3)
Recent travel and/or recent fever	1,173	20	20/23 (87.0)
All people	5,520	23	23/23 (100.0)

## Discussion

The primary risk factor for malaria infection in the low-transmission district of Richard Toll is travel. Most people (80%) with a malaria infection identified through passive case detection reported travel in the previous two weeks. RDT-positive prevalence among family and neighbours of the index case was significantly lower among those who reported no recent travel (0.2%) compared with those who reported travel within the previous 15 days with variations on risk based on location of travel – including travel within the Saint-Louis region (3.1%), Dakar region (10.5%), and other regions of Senegal (33.3%). Index malaria cases were disproportionately male (82%) and infection was more common among male family/neighbours (0.6%) compared with females (0.2%). Although confirmation through additional and longer term surveillance methods is needed, results from this 12-week pilot are consistent with either no or very low levels of local malaria transmission in the Richard Toll district of Senegal and infections appear to be acquired primarily during travel. With marked malaria transmission reduction, burden commonly shifts from young children and pregnant women to adult men with occupational or behavioural factors that put them in contact with infectious mosquitoes [7]. In other contexts, this has included men who migrate to higher risk areas for work, including forest and plantation work [16]. Results from a recent case control study in Ethiopia suggest that travel can be a risk factor for malaria infection even when it occurs within areas considered to be endemic [19].

Intervention and monitoring strategies are needed to effectively target this population of travellers residing in northern Senegal. Reduction of malaria transmission in areas within Senegal to the south of Richard Toll may be the most important factor in reducing malaria infections in Richard Toll as it is travel to these areas that is apparently the risk that reintroduces malaria to the district. Until this malaria transmission reduction is achieved, intervention strategies targeting travellers could include specific communication to use ITNs for prevention when travelling and to seek prompt evaluation and treatment for suspected malaria. Promoting prevention and treatment among this population may be challenging given that many infections will be asymptomatic. Clearly, the index cases were identified based on presentation with fever to a health worker who then tested the case and identified the infection. The finding of additional infections among household members or neighbours must go beyond simple screening for history of fever. On the one hand, asking household and neighbours about recent fever was helpful in identifying a group with increased risk of malaria infection, yet most RDT-positive family and neighbours of index cases reported no current or recent fever (70% asymptomatic). Importantly, a history of recent travel was also helpful in identifying cases. People who frequently travel can be identified – for example, people travelling for school or work in certain occupations, and they can be targeted for blood testing and treatment to clear any identified infections. As malaria transmission has dropped dramatically in northern Senegal, past methods of identifying and monitoring at-risk populations (e.g., young children and pregnant women [1]) are no longer useful for tracking transmission. Alternatively, measuring prevalence among the newly recognized population at risk through more targeted surveys could provide relevant malaria burden estimates.

### Optimizing case investigation

The success of malaria case investigations for transmission reduction will be facilitated by operations that are both effective in identifying all (or nearly all) additional cases and efficient with human and financial resources. The Richard Toll case investigation experience can be used to optimize the efficiency of this strategy. The overall effort tested 5,520 individuals and identified 23 infections – a lot of work for a few cases. Similar to findings in Swaziland [13], a substantial fraction of infections (14/23) were located within the index case compound. Thirteen infected individuals reported recent travel, and seven reported recent fever. Modification of the case investigation strategy could include selective testing limited to people: 1) residing with the index case compound; 2) neighbours with any recent travel; and, 3) neighbours with any recent fever. This strategy would have identified 20 (87%) of the 23 infections by screening 1,173 individuals – 79% fewer people tested. Information on the remaining three infections suggests that screening for boarding school students would have identified all cases – as the three remaining cases were males attending secondary school or university away from home. Although additional pilot evaluation is needed, school enrolment away from home may serve as a useful screening question for identifying infections. Locally developed evidence to shape reactive case detection components of case investigation is needed [16]; this study highlights the need to incorporate demographic risk factors in screening during reactive case detection [7]. More specific testing criteria in this context will save time and resources that would otherwise be spent on unnecessary malaria blood testing.

The current experience in northern Senegal must be taken to scale and evaluated once more to confirm the gain in efficiency. This study reports findings from the pilot phase of a programme during which demographic factors were identified to optimize the investigation protocol. The pilot was characterized by substantial investment in resources; investigation staff were identified from community, facility, district, national, and control programme partner levels, and all levels were equipped with resources necessary for field investigations. Detailed data on all index cases, family, and neighbors were collected and analysed to guide scale-up. When taken to scale, efforts to cost the programme and identify efficiencies at scale will be needed. It will also be critical to monitor operations and identify strategies to maintain high rates of individual participation as well as timely investigations and follow-ups. Information to optimize continued roll-out and scale-up reported here strengthens an initial foundation for this surveillance system component. Continued systems strengthening to ensure a functional, effective, and efficient operation at scale will be needed.

The Richard Toll pilot treated RDT-positive individuals according to national policy using artemether lumefantrine (AL). This artemisinin combination therapy (ACT) is appropriate for control in contexts where re-infection is likely. An elimination approach may consider employing longer acting drugs that clear infection and provide some duration of prophylaxis to further prevent transmission [7,20]. Applications in this context could entail using dihydroartemisinin piperaquine – an ACT with a post-treatment prophylactic effect that is longer than other ACT, including AL [21]. An elimination approach may also include using a gametocytocidal drug such as a single low dose of primaquine recently recommended by WHO for use in combination with ACT for all patients with parasitologically confirmed *Plasmodium falciparum* malaria [22]. Infection management could further be optimized by using a single dose effective treatment; although not currently available, a single dose treatment could be given as directly observed therapy to optimize effectiveness [20].

Improved diagnostics are another area for optimization in case investigation activities. This pilot used RDTs for reactive case detection. RDTs may miss infections detectable by more sensitive methods [17], and submicroscopic infections may contribute to transmission in such settings [18]; thus further evaluation of more sensitive diagnostic tests for case investigation is warranted. At present, the most sensitive diagnostic tests are not easily deployed in field settings. Diagnostic tools that are field-ready and capable of detecting low parasite densities may be needed to improve community-based surveillance [7,23], however, within this pilot effort, no emerging malaria infections were observed that had been missed by RDT testing in the initial investigation. In the absence of field-ready tools, attempts to deploy PCR in the field have been made in the context of intensive activities to control spread of artemisinin-resistant parasites in western Cambodia [24]. The artemisinin resistance containment project deployed PCR for cross-sectional community screening and follow-up and treatment within high-incidence villages. The extent to which employing PCR for cross-sectional community screen and treat work could successfully be applied to ongoing case investigation programmes is unclear.

An alternative for halting transmission among malaria-affected households is the mass drug administration (MDA) approach whereby all household members are treated in lieu of any testing. While this is certainly an option to block transmission, this approach fails to provide any information on infection and precludes analyses that could identify extent of and risk for infection. Initial case investigation work benefits from gathering substantial data on infection and risk factors for infection as this information can inform protocol modifications for improved efficiency.

### Study limitations

This 12-week pilot gathered substantial information from index cases and family and neighbours to understand patterns of risk and infection in a low-transmission setting. Limitations of the information gathered during the pilot include the limited scale and time frame. The activity targeted one district, and implementation occurred at a sub-district level due to the national data retention strike. Despite this limited scale, case investigation activities in this area were timely given the context, where recent routine and population-based survey data had indicated very low levels of transmission. Additionally, the pilot was an avenue for exploring risks in Richard Toll district that are likely relevant in neighbouring districts in northern Senegal. The duration of the pilot project was limited; however time was sufficient to generate enough cases and investigations to begin to draw conclusions about risk factors in this context. It is possible that risk factors identified during a limited time frame may not be generalizable to other time periods in the same context. However, the pilot period encompassed peak transmission season, thereby facilitating identification of the most critical risk factors for infection. While there is no reason to believe that the specific time period of this pilot introduced any bias in results, findings should be validated as case investigation strategies are expanded and implemented year-round.

RDTs were a practical tool for point-of-care testing and household and neighbourhood investigation in this pilot. However, more sensitive assays (PCR) would likely identify additional infections. While RDTs may be imperfect, there is no evidence to suggest that individuals who are RDT-negative but harbor a transmissible infection detectable by PCR will differ on the characteristics shown here to be associated with infection. Nonetheless, use of molecular methods to identify all infections would strengthen conclusions on risk factors for infection. Future studies using highly sensitive assays as an evaluation tool can further examine the frequency and transmission potential of RDT-negative but sensitive-assay-positive individuals.

The data collection tool used for this study assessed potential risk factors for infection, including age, sex, occupation, recent travel, and bed net use. It is possible that specific relevant risk factors were not measured because they have not yet been identified. Nonetheless, the risks that were investigated and identified in this study make sociologic sense and lend themselves to action. Travel was identified as an important risk factor, including travel to Dakar and other regions in Senegal. However, data collected in this study are unable to pinpoint the source of infection and should not be used to draw conclusions about specific geographic areas for malaria transmission in Senegal.

### Evidence gaps

Travel is clearly a risk for infection; however this study provided insufficient evidence to effectively target travellers at risk for prevention, screening and treatment work. Further work is needed to better define this population at risk, the nature of the risk that they face, and strategies to mitigate risk of infection and treat infections. Recent advances and applications of mobile phone data offer possibility to map mobility and malaria infection risk [25].

This activity identified potential for introducing relevant screening questions that would reduce human and financial resources needed for reactive case detection. The next iteration of case investigation in northern Senegal should include careful monitoring of such strategies. The work in Richard Toll district operated in the context of a national data retention strike whereby public health workers were withholding routine monitoring data – such as malaria case data – from national health information systems. Subsequently, the pilot activity was limited to a sub-district scale. There is need to monitor and document lessons learned for effective and efficient case investigation at larger scale – including the entire district and expansion to other low-transmission districts in northern Senegal. Evidence from other contexts is needed to build an evidence base around efficient and effective strategies for case investigation. The evidence agenda around case investigation programmes taken to scale should also incorporate measures to track impact on malaria transmission and prevention of reintroduction.

## Conclusions

Practical solutions for effective and efficient surveillance are required as countries progress towards malaria elimination. This pilot study demonstrates the feasibility of introducing and then improving case investigation and reactive case detection in a low-transmission setting in northern Senegal. Expanding and optimizing case investigation with specific and efficient targeted testing and treatment of at-risk family and neighbours strengthens the systems needed for continued progress towards malaria elimination in this setting. Optimization in this context can be achieved through focusing first on index-case-households and limiting neighbour testing to those who report recent fever or travel. Additional interventions to reduce the risk of malaria infection among travelers are also needed in this region.

## Competing interests

The authors declare that they have no competing interests.

## Authors’ contributions

RWS, DE, MB, and YD conceived and designed the programme work and evaluation. GDS, AN, MB, BMM, YD, and BM were responsible for case investigation work. ML analysed the data and wrote the first draft of the manuscript. ML, GDS, AN, MB, BMM, YD, BM, DE, and RWS contributed to writing of the manuscript. All authors read and approved the final version of the manuscript.
